# Lingual cavernous hemangioma in a Nepalese boy—‘A Difficult Associate!!!’

**DOI:** 10.1093/jscr/rjy283

**Published:** 2018-10-23

**Authors:** Ashish L Shrestha, Shova Banstola Paudel

**Affiliations:** 1 Department of Pediatric Surgery, Grande International Hospital, Dhapasi, Kathmandu, Nepal; 2 Department of Pathology, Grande International Hospital, Dhapasi, Kathmandu, Nepal

## Abstract

Hemangiomas are benign tumors comprising of ectatic blood vessels. Although common in the head and neck region, those occurring within the oral cavity and tongue are exceedingly rare. We report a 5-year-old boy with a swelling over the anterior third of tongue post failed conservative therapy eventually treated with surgical excision and confirmed histologically as a cavernous hemangioma probably first of its kind reported from Nepal.

## INTRODUCTION

Based on Mulliken and Glovacki Classification (1982), vascular lesions are categorized broadly into two types: hemangiomas and vascular malformations, former being the commonest lesions of infancy and childhood. Hemangiomas are classified based upon histology into capillary and cavernous types and upon vessel involvement into high flow (arterial and arteriovenous) and low flow types (capillary or venous) [1].

Most hemangiomas of head and neck appear few weeks post natally, attain rapid growth, enter a phase of involution and eventual spontaneous resolution by 5–8 years [2]. However, in some they may persist and pose treatment challenges. We hereby present a boy who had a persistent and expanding lingual hemangioma presenting with macroglossia despite attempted conservative measures.

## CASE REPORT

A 5-year-old boy presented with an unsightly and protuberant swelling over the anterior third of tongue that had started as a small nodule soon after birth and gradually progressed in size after 9 months of age. It was associated with difficulty in swallowing, speech, recurrent blistering with pain and bleeding with minor trauma. He was unable to contain it within his mouth necessitating to sleep open-mouthed. He had also developed multiple dental caries with it.

**Figure 1:**
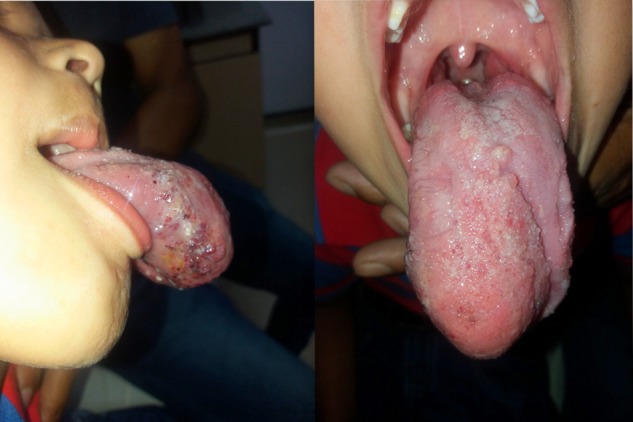
Appearance of the lingual cavernous hemangioma on clinical examination.

He had been managed conservatively with beta-blockers and steroids elsewhere for years with no appreciable benefit. General examination was normal. Oral examination revealed a well demarcated swelling involving the anterior third of tongue extending dorso-ventrally (occupying mostly the ventral surface) and measuring 6×5×3 cm^3^, red in color with a purplish hue. It had a smooth surface, with fine granularity. It was partially compressible and soft in consistency as shown in Fig. 1. Also noted were multiple dental caries. Examination of the neck and rest of the systems was unremarkable.

**Figure 2:**
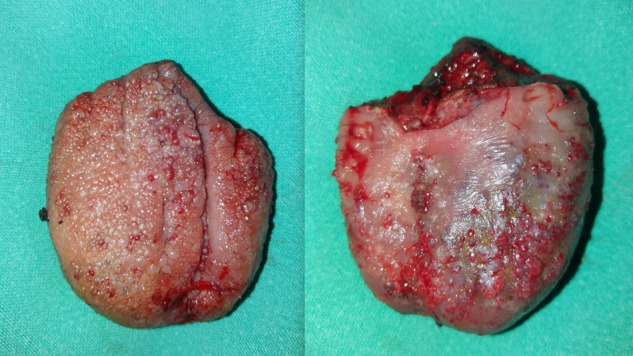
The dorsal and ventral appearance of the lingual hemangioma immediately after excision.

A working clinical diagnosis of macroglossia secondary to lingual hemangioma was made and partial glossectomy using an inverted V incision was performed as shown in Fig. 2.

**Figure 3:**
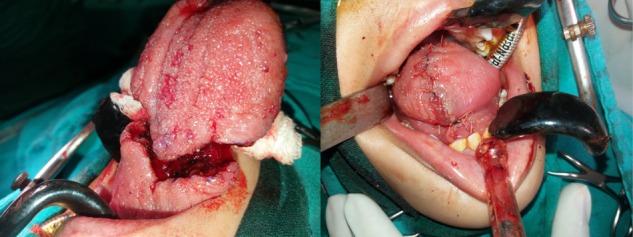
On table appearance of the specimen nearing excision and after reconstruction of the residual tongue.

The limbs of V were approximated to reconstruct the residual tongue as shown in Fig. 3

**Figure 4:**
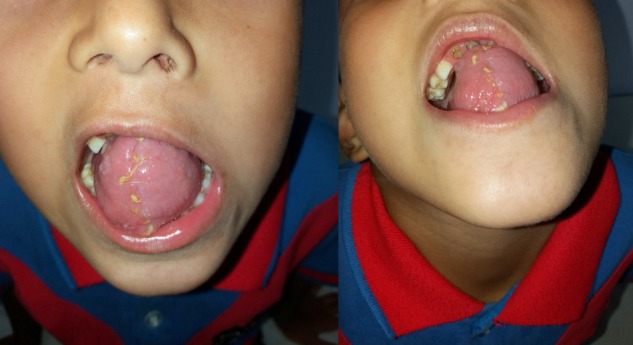
Dorsal and ventral appearance of the reconstructed residual tongue at outpatient follow up after 10 days.

At 10 day outpatient follow up, improved swallowing, speech clarity and good healing was observed as shown in Fig. 4.

**Figure 5:**
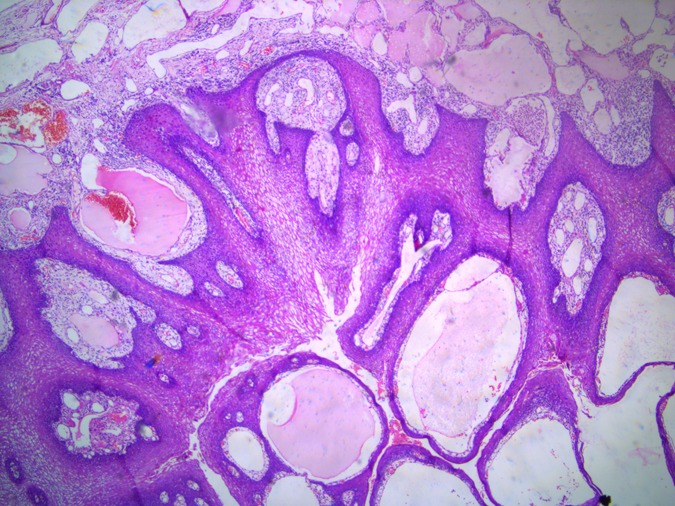
Microscopic appearance of the lingual cavernous hemangioma stained with eosin and hematoxylin at four times magnification.

**Figure 6:**
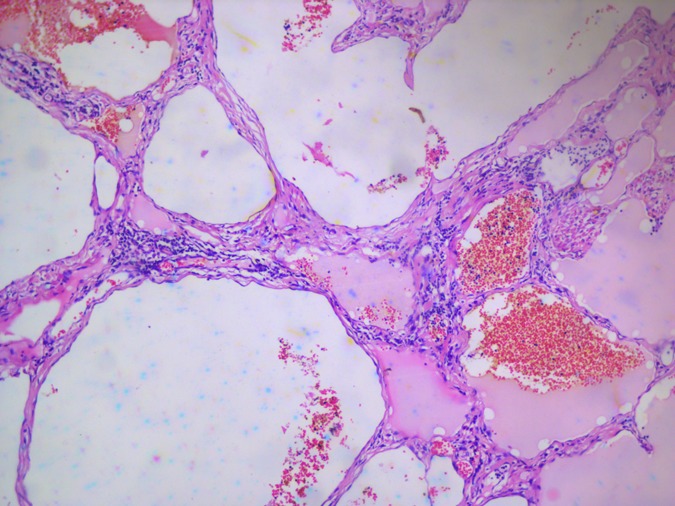
Microscopic appearance of the lingual cavernous hemangioma stained with eosin and hematoxylin at 10 times magnification.

The histopathology report showed mucosa lined by stratified squamous epithelium, with underlying stroma showing dilated vascular channels lined by endothelial cells and containing red blood cells in their lumen confirming the diagnosis of lingual cavernous hemangioma as shown in Figs 5 and 6.

## DISCUSSION

Of the majority of hemangiomas that tend to occur in the region of head and neck, the ones that occur in the tongue deserve special consideration not merely due to the rarity of occurrence but also due to associated issues with swallowing, breathing, susceptibility to trivial trauma and cosmesis [3]. Like other hemangiomas most of these glossal lesions appear 2–4 weeks after birth, growing rapidly till 6–8 months age and then following a usual course of involution in 70% of patients to a state of near disappearance by 5–8 years [3]. Less commonly, these lesions may be associated with Sturge–Weber syndrome, Osler–Weber–Rendu syndrome and Blue rubber bleb nevus syndrome [3]. The characteristic history and local examination findings aided by histopathological studies confirms the diagnosis in most cases. The treatment modalities include watchful observation, intralesional and systemic steroids, beta blockers, embolization, sclerotherapy and surgical excision [3]. The choice of therapy depends upon factors like age, size, location, lesion hemodynamics and response to the modality used [2, 3]. Having treated earlier with systemic steroids and beta blockers with unsuccessful outcome, we opted to proceed with surgical excision for our patient with a successful and satisfactory outcome.

## References

[1] Kripal K, Rajan S, Ropak B, Jayanti I Cavernous hemangioma of the tongue. Case Rep Dent 2013;2013:898692. 24073342 10.1155/2013/898692 PMC3773376

[2] Pranitha V, Puppala N, Deshmukh SN, Jagadesh B, Anuradha S Cavernous hemangioma of tongue: management of two cases. J Clin Diagn Res;2014 8(10):ZD15–17. 10.7860/JCDR/2014/10216.5005 PMC4253281 25478463

[3] Khanduri S, Agrawal D, Varshney G, Singh N Haemangioma of tongue: a rare case report. JOOMR 2015;3:25–7.

